# Petri-net-based 2D design of DNA walker circuits

**DOI:** 10.1007/s11047-018-9671-4

**Published:** 2018-02-28

**Authors:** David Gilbert, Monika Heiner, Christian Rohr

**Affiliations:** 1 Brunel University London, Uxbridge, UB8 3PH UK; 2 Brandenburg Technical University Cottbus-Senftenberg, Postbox 10 13 44, 03013 Cottbus, Germany

**Keywords:** Stochastic Petri nets, Coloured Petri nets, DNA walker systems, Design assessment, Leakage transitions, Structural analysis, Qualitative analysis, Stochastic analysis, Simulative model checking

## Abstract

We consider localised DNA computation, where a DNA strand walks along a binary decision graph to compute a binary function. One of the challenges for the design of reliable walker circuits consists in leakage transitions, which occur when a walker jumps into another branch of the decision graph. We automatically identify leakage transitions, which allows for a detailed qualitative and quantitative assessment of circuit designs, design comparison, and design optimisation. The ability to identify leakage transitions is an important step in the process of optimising DNA circuit layouts where the aim is to minimise the computational error inherent in a circuit while minimising the area of the circuit. Our 2D modelling approach of DNA walker circuits relies on coloured stochastic Petri nets which enable functionality, topology and dimensionality all to be integrated in one two-dimensional model. Our modelling and analysis approach can be easily extended to 3-dimensional walker systems.

## Introduction

DNA computing building on DNA strands (molecules) interacting by DNA strand displacement (DSD) is a research focus in computer science and nanomedicine alike (Boemo et al. 2016). DSD can be thought of as a formal computing language (Phillips and Cardelli 2009) for the engineering of DNA-only chemical controllers, sensors, etc. (Chen et al. 2013). Two DSD categories can be distinguished (Boemo et al. 2016). (1) In *floating DNA systems*, DNA strands are freely moving molecules in a well-mixed solution; i.e., there are no geometric constraints preventing two molecules from interacting. (2) *Localised DNA systems* impose constraints by tethering DNA strands (anchorages) to a rigid lattice, forming a DSD circuit. An additional DNA strand (walker) may move along the lattice organised in origami tiles, thus performing a computation, e.g., by walking along a binary decision tree, possibly reduced to a directed acyclic graph (DAG), yielding a *binary decision DAG*, in the following briefly called DAG. Different options for programming a given DSD circuit are known to force a walker to follow a specific path (Boemo et al. 2016).

There are a couple of challenges for the design of reliable DSD circuits. DSD circuits are inherently undirected, and thus do not directly encode DAGs. A walker may take a shortcut or even jump into another path; the latter is known as a leakage transition. Therefore, the experimental design of DSD circuits clearly calls for tool support.

Floating systems are supported by the Microsoft Visual DSD tool (Lakin et al. 2011), while localised systems are considered in Dannenberg et al. (2015) and Barbot and Kwiatkowska (2015); none supports the automated identification of leakage transitions. Modelling DNA computing devices with freely moving molecules closely resembles modelling approaches for chemical reaction networks as they are widely used in systems and synthetic biology; e.g., we could deploy Petri nets as umbrella language opening the doors to qualitative, stochastic and deterministic analysis techniques, as we have previously demonstrated in Gilbert et al. (2007), Heiner et al. (2008) and Blätke et al. (2015).

### Contributions

In this paper we consider localised DNA computation. We start from the modelling approach for walker circuits introduced in Dannenberg et al. (2015) and represented as stochastic Petri nets in Barbot and Kwiatkowska (2015), with the purpose of stochastic analysis to assess the reliability of the circuit design. To assist the circuit designer by a more detailed assessment, we refine the stochastic analysis, complemented by merely qualitative, and thus computationally less expensive analyses. More specifically we discuss the automated identification of leakage transitions and how to quantitatively compare different circuit designs for a given DAG. Leakage can be reduced by employing a circuit layout topology that optimises the distance between any two anchorages to avoid potential leakage transitions, and which in general can be achieved by increasing the area of the circuit for a given size (in terms of number of anchorages).

However, one goal of DNA circuit design is in fact to minimise the circuit area (Jung et al. 2015). Thus the ability to identify leakage transitions is an important step in the process of optimising DNA circuit layouts where the aim is to minimise the computational error inherent in a circuit while minimising the area of the circuit. This trade-off is quantified by a combination of structural and probabilistic analysis techniques including performability measures building on impulse rewards.

Moreover, we show how coloured Petri nets can be used to obtain a generic template for specifying DNA walker systems, while preserving the ability of a mathematically rigorous assessment of the system specification. This template may be easily adjusted to different stepping scenarios or distance notions without requiring programming skills. In this paper, we consider 2-dimensional walker systems. However, the extension of our flexible modelling approach to the 3-dimensional case is straightforward.

### Outline

In the next section we discuss the modelling of DNA walker circuits, first as planar undirected graphs, which we convert into Petri nets to be able to analyse their execution, and finally into coloured Petri nets to obtain a concise and flexible circuit specification incorporating 2D topology information. Afterwards we introduce in Sect. 3 our new technique to identify leakage transitions, followed by a brief overview on Petri net related analysis techniques with a special focus on stochastic analyses in Sect. 4. We demonstrate the usability of our techniques by comparing two layouts for a given DNA walker circuit taken from Dannenberg et al. (2015). We conclude our paper in Sect. 5 with a brief summary and outlook on future work.

## Modelling

### DNA walker systems

We consider programmable DNA walker circuits introduced in Yin et al. (2004), Bath et al. (2005), Wickham et al. (2011) and Wickham et al. (2012), which are known to exhibit an inherently probabilistic behaviour. DNA walker circuits have been modelled and analysed with the PRISM tool (Dannenberg et al. 2015; Dannenberg 2016), and later by help of stochastic Petri nets (Barbot and Kwiatkowska 2015). To be self-contained we recall the basic facts required to understand our modelling approach deploying coloured stochastic Petri nets.

**Fig. 1:**
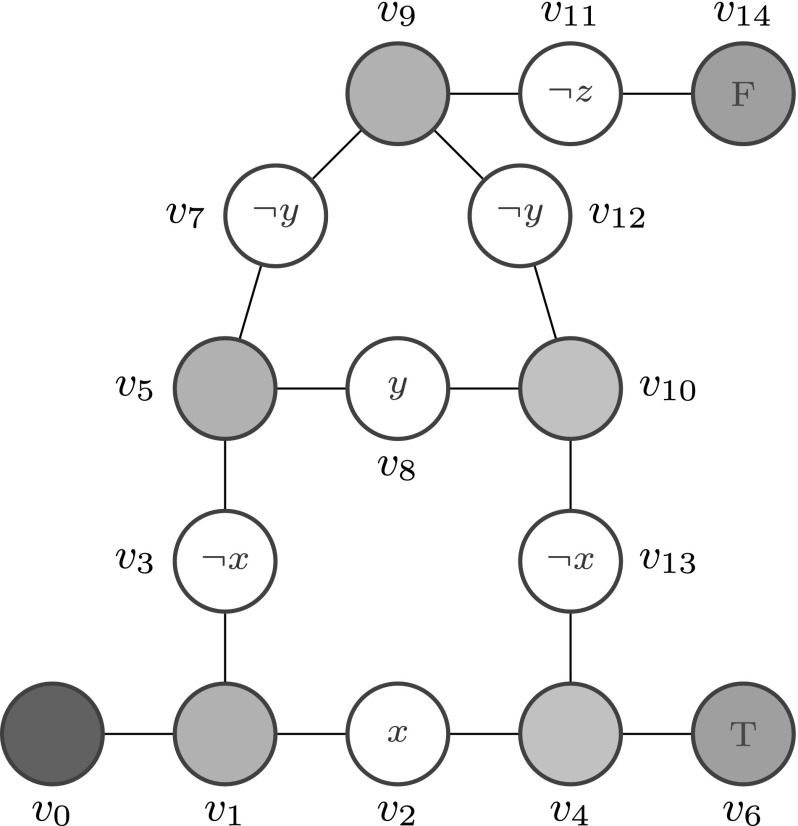
DSD graph representing the boolean function $x\vee y\vee z$. Colour code: blue—INIT, green—FORK, orange—JOIN, red—FINAL; uncoloured—NORM; $\epsilon$ label not shown. (Color figure online)

The DNA walker circuits under consideration are supposed to compute a Boolean function over *n* input variables, i.e., ${\mathcal {B}}^{n} \rightarrow {\mathcal {B}}$. Formally, a DNA walker circuit defines a planar *undirected graph*, in the following called DSD graph; see Fig. 1 for an example. Vertices stand for anchorages and undirected edges for possible walker steps. Vertices with two adjacent edges form linear tracks of the walker circuit, while vertices with three adjacent edges represent gates, i.e., either forking or joining junction points. There are no vertices with more than three adjacent edges, because there is a lack of experimental evidence (Wickham et al. 2011, 2012).

Anchorages may be labelled with literals over the domain of the Boolean function to be evaluated. The programming of the circuit is achieved by blocking those anchorages whose labels are evaluated to false for the given input values. These observations are summarised in the following definition which builds on the one given in Dannenberg (2016).

#### **Definition 1**

(*DSD graph, syntax*) A DSD graph is a tuple ${\mathcal {G}}= ({V,E,In,L,{ Out}})$, where

- *V* is the set of vertices, with
  $$V= V_{{ INIT}} \cup V_{{ NORM}} \cup V_{{ FORK}} \cup V_{{ JOIN}} \cup V_{{ FINAL}}$$, and
  - $V_{{ INIT}} = \{v_0\}$—the unique initial anchorage,
  - $V_{{ NORM}}$—the vertex set of normal anchorages,
  - $V_{{ FORK}}$—the vertex set of fork anchorages,
  - $V_{{ JOIN}}$—the vertex set of join anchorages,
  - $V_{{ FINAL}}$—the vertex set of final anchorages,
  and all vertex sets pairwise disjunctive.
- *E* is the set of undirected edges with $E \subset \left( V \times V\right)$. The initial vertex $v_0$ and final vertices have one adjacent edge, normal vertices have two adjacent edges, and junction vertices (either fork or join) have three adjacent edges.
- ${ In}$ is a set of Boolean variables, and ${ literals}(In)$ yields the set of all value assignments over *In*.
- *L* is a labelling function with $L{:} V {\setminus } V_{{ FINAL}} \rightarrow { literals}(In) \cup \{\epsilon \}$.
- ${ Out}$ is an output function with ${ Out}: V_{{ FINAL}} \rightarrow \{T,F\}$, assigning a truth value to each final vertex. □

#### *Remarks*

- All undirected edges have exactly one direction which corresponds to a step directed from the init vertex to one of the final vertices.
- By definition, the walker can never leave a final node; however this cannot be deduced from the undirected graph.
- Binary decision trees do not require join anchorages.
- There has to be at least one final vertex. Usually the graph will contain at least two final vertices, with each truth value occurring at least once.
- DSD graphs with exactly one final vertex labelled with *true* allow for composability, e.g., exploiting origami tiles.
- The empty label $\epsilon$ permits unblockable anchorages and is typically not displayed. The unique initial anchorage $v_0$ should be labelled with $\epsilon$.
- The definition given in Dannenberg (2016) does not distinguish between fork and join, and it assigns ${ literals}(in)$ to edges; we assign them to vertices.

A DSD graph may be seen as a finite automaton. It describes a map with all possible steps a DNA walker may take to execute the computation encoded in the underlying (binary decision) DAG for any input values. Accordingly, for a given set of input values, a walker is supposed to go only to anchorages, where the evaluation of the label yields *true*. The anchorages where the evaluation yields *false* are considered to be blocked; thus can not be visited by the walker. Assuming a consistent labelling, for each possible set of input values, there exists a path from the initial to a final vertex delivering the result. If there exists exactly one path, we call the DSD graph *deterministic* (Dannenberg 2016).

The DNA walker starts its journey at the unique initial vertex, and then follows one of the adjacent edges to reach a neighbouring unblocked vertex, which is repeated until reaching a final vertex, which by definition can not be left again. The final vertex reached indicates the result computed by the walker’s journey through the DNA circuit.

Undirected edges can be read as a shorthand notation for two opposite, directed edges; these two directed edges stand for possible walker steps in opposite directions. Thus, a DNA walker does not go on a target-oriented journey; it can not distinguish between fork and join anchorages, and all anchorages reachable in one step have the same probability to be visited next. For example, assuming $x={ true}$ in Fig. 1, a walker may repeatedly move along $v_{0}-v_{1}-v_{2}-v_{4}$ (in both directions), before accidentally finding the final vertex $v_{6}$. To put it differently, the challenge consists in realising an algorithm working on a directed graph (the DAG) by use of an undirected graph (the DSD graph).

A DNA walker’s life becomes slightly easier in “burnt-bridges” circuits, where each position can only be visited once. As already visited positions are not among the possible choices of target positions for the next step, the walker will generally be driven in the direction of a final vertex.

This execution semantics goes beyond standard graph-based reasoning and is not covered by Definition 1. To formalise the execution semantics, we convert the DSD graph into a Petri net—first into a plain Petri net, inspired by the approach introduced in Barbot and Kwiatkowska (2015), and afterwards into a coloured Petri nets, which will yield a concise template for DNA walker circuit specifications.

#### Stepping distance

It has been observed that a walker may move in one step to all unblocked anchorages within a certain radius, but with different probabilities (Wickham et al. 2011). We employ the approximation reported in Dannenberg et al. (2015) and assume that the walker stepping rate *k* is a piecewise function of the distance *d* over the maximum interaction distance $d_M$, the average distance between anchorages $d_{a}$, and the base rate $k_{s}$, given by:

1 $$k = {\left\{ \begin{array}{ll} k_s & \quad {\text {if}}\quad d \le 1.5 \cdot d_a\\ k_s/50 & \quad  {\text {if}}\quad 1.5\cdot d_a< d \le 2.5 \cdot d_a\\ k_s/100 & \quad {\text {if}}\quad 2.5\cdot d_a < d \le d_M\\ 0 & \quad \text {else}, \end{array}\right. }$$

with $d_M = 24\,{\hbox {nm}}$, $d_a = 6.2\,{\hbox {nm}}$, $k_s = 0.009\,{\hbox {s}}^{-1}$. This will generally add further unintentional undirected edges to the DSD graph; how many and which ones depends on the topology.

### Petri nets

To be self-contained we briefly recall basic Petri net concepts, which will allow us to formally treat the execution semantics of DSD graphs; for more details see Heiner et al. (2008).

#### **Definition 2**

(*Petri net, syntax*) A Petri net is a tuple ${\mathcal {N}}=\left( P,T,f,m_0\right)$, where

- *P* and *T* are finite, non-empty, and disjoint sets. *P* is the set of *places*, and *T* is the set of *transitions*.
- $f:\left( (P\times T\right) \cup \left( T\times P\right) )\rightarrow N_0$ defines the set of directed *arcs*, weighted by non-negative integer values.
- $m_0:P\rightarrow N_0$ gives the *initial marking*. □

The pre-set of a node $x \in P \cup T$ is defined as ${\scriptstyle ^\bullet }x:=\left\{ y\in P \cup T|f\left( y,x\right) \not = 0\right\}$, and its post-set as $x{\scriptstyle ^\bullet }:=\left\{ y\in P \cup T|f\left( x,y\right) \not = 0\right\}$. We extend both notions to a set of nodes $X \subseteq P \cup T$ and define the set of all pre-nodes ${\scriptstyle ^\bullet }X := \bigcup _{x\in X} {{\scriptstyle ^\bullet } x}$, and the set of all post-nodes $X{\scriptstyle ^\bullet } := \bigcup _{x\in X} {x {\scriptstyle ^\bullet }}$.

#### **Definition 3**

(*Petri net, semantics*) Let ${\mathcal {N}}=({P,T,f,m_0})$ be a Petri net.

- A transition *t* is *enabled* in a marking *m*, written as $m[t\rangle$, if $\forall p \in {\scriptstyle ^\bullet } t: m(p) \ge f(p,t)$, else *disabled*.
- A transition *t*, which is enabled in *m*, may fire.
- When *t* in *m* *fires*, a new marking $m'$ is reached, written as ${m} {\xrightarrow {\text {t}}} {m'}$, with
  $$\begin{aligned} \forall p \in P : m'(p) = m(p) - f(p,t) + f(t,p). \end{aligned}$$
- The firing happens atomically. □

In qualitative (time-free) Petri nets, the firing does not consume any time, while in stochastic Petri nets, transitions are associated with generally state-dependent firing rates. The repeated firing of enabled transitions (the game) yields the behaviour of a Petri net. Generally, there are more than one transition enabled in a given marking. Then the decision of the transition to fire next is taken non-deterministically in time-free Petri nets, and in accordance with the stochastic firing rates in stochastic Petri nets.

**Fig. 2:**
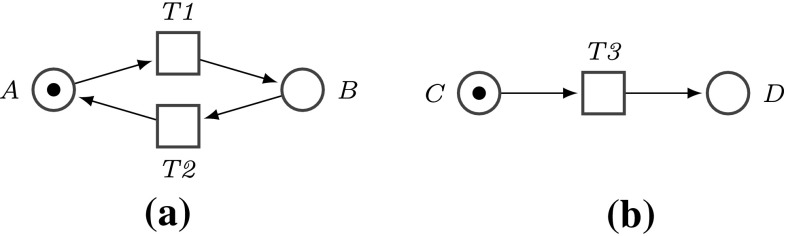
DNA walker basic stepping scenarios, with *A*, *B*, *C* non-final vertices, and *D* final vertex. **a** Standard step, **b** final step

Transforming a DSD graph into a Petri net is straightforward: the vertices are turned into Petri net places and directed edges into Petri net transitions, such that the source and sink vertex of a given edge become the pre- and post-place of the corresponding transition, see Fig. 2. We keep the terminology introduced for DSD graphs and speak of init/norm/fork/join/final places. Finally, we model the DNA walker by a token which we set on the init place. Now, playing the token game will produce all possible paths (of arbitrary length) a walker can take for any input values, which will sooner or later end in a final place. The system behaviour has reached an intended dead state (no transition is enabled).

**Fig. 3:**
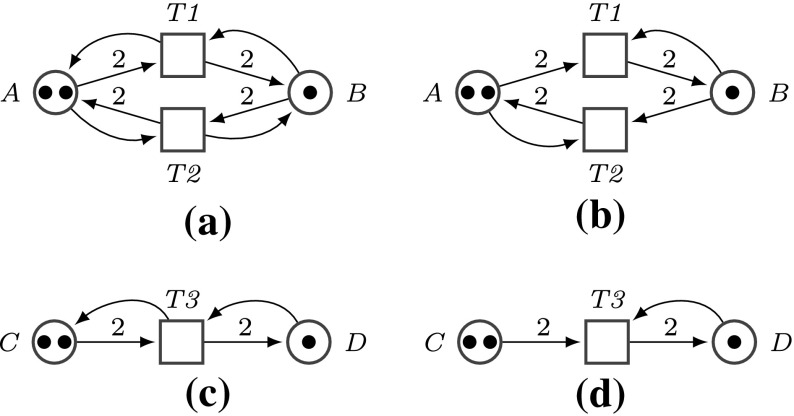
DNA walker stepping scenarios, with *A*, *B*, *C* non-final vertices and *D* final vertex. In this paper, we focus on the “burnt-bridges” setting. **a** Unguided step, **b** “burnt-bridges” step, **c** unguided final step, **d** “burnt-bridges” final step

To control, how often a place can be visited, we adopt the modelling idea introduced in Barbot and Kwiatkowska (2015), compare Fig. 3. Initially all unblocked places hold one token, indicating that the place can be visited, and the init place holds additionally a token representing the walker. Then, a directed edge of the DSD graph going from vertex *A* to vertex *B* is modelled by a Petri net transition, which checks if the target place *B* can be visited (is not blocked). When a walker moves from place *A* to place *B*, then in total for

- *Unguided scenario*: one token is removed from *A*, because the walker leaves *A*, and one token is kept, because *A* can be re-visited,
- “*Burnt-bridges” scenario*: two tokens are removed from *A*, because the walker leaves *A*, and *A* can not be re-visited.

In both cases, a second token is added to *B*, because the walker is now on *B*. In these scenarios, playing the token game will produce a path the walker takes, which typically goes straight to a final place (assuming a consistent labelling). This path will be unique for deterministic DSD graphs. By repeatedly re-initialising the Petri net we can explore all possible paths for any input values, which will be systematically done in the next section.

In summary, the conversion of a DSD graph into a Petri net is rather flexible and can be conveniently adjusted to the particular execution semantics on hand. In the following we focus on the “burnt-bridges” scenario, which is also easier to implement in DNA than in the “unguided” approach (Bath et al. 2005).

#### Stepping distance

So far, our Petri net model only contains those steps that a walker may take to follow a path in a given DSD graph. If the DSD graph were to be directed, these steps would all be intentional.

The walker stepping rate *k* (see Eq. 1) introduces three categories of steps: short distance, medium distance and long distance steps. Ideally a spatial layout of a walker circuit should ensure that all short distance steps correspond to edges in the DSD graph. The number of additional medium and long distance steps obviously depends on the topology. We discuss the influence of the topology on the number of steps in each category in Sect. 3.

These additional steps may introduce unwanted behaviours. Now, a walker can take shortcuts along a path, jump backwards in the path just taken, or jump to another branch, known as leakage steps (transitions). As a result, a walker can get lost in a non-final vertex without any neighbouring vertex free to be visited; technically speaking—the system behaviour may reach an unwanted dead state. To be able to distinguish between wanted and unwanted dead states, we add a loop (i.e.,a transition having the same pre- and post-place) to all places modelling final vertices. The number of leakage transitions and dead states will have an influence on a circuit’s reliability, which we quantify in Sects. 3 and 4.

#### Fault model

**Fig. 4:**
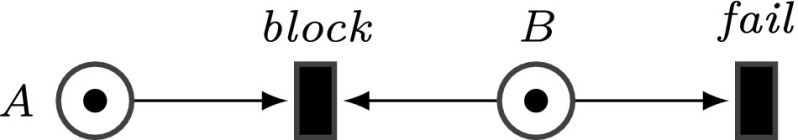
Fault model added for every anchorage (place) *A* to be blocked. The success rate of *block* is assumed to be $f=0.7$ and of *fail* $f=0.3$

The programming of a walker circuit according to the given input values of the Boolean function to be computed is realised by the blocking of the correspondingly labelled anchorages. This blocking mechanism may fail. To reflect this we follow the approach introduced in Barbot and Kwiatkowska (2015) and add a fault model to the Petri net we obtained so far, modelling the failure of the blocking mechanism; see Fig. 4.

In order to simplify the modelling, we now assume that all places hold initially a token. For the anchorages to be blocked, this token is removed by additionally added blocking transitions. However, the firing of blocking transitions can be prevented by transitions in conflict, representing the occurrence of a failure. If a failure transition fires, an anchorage to be blocked remains unblocked, and thus a walker can move to this anchorage and follow an incorrect path.

As the programming has to happen before the walker reaches a junction, transitions representing the blocking mechanism and its potential failure are modelled as *immediate transitions*, i.e., transitions which fire without any time delay and highest priority, thus before any stochastic transition will fire; see Heiner et al. (2009) for details. We assume a uniform failure of the blocking mechanism; thus all pairs of immediate transitions are equally weighted with a probability of ${\hbox {f}} = 0.7$ for the blocking transitions, and a probability of ${\hbox {f}} = 0.3$ for the failure transitions.

### Coloured Petri nets

Colouring yields a form of high-level Petri nets which permit the description of similar network structures in a concise way using colours grouped in colour sets—to be understood as a synonym for discrete data types as known from programming languages. The colouring principle can be equally applied to qualitative and quantitative Petri nets (Blätke et al. 2015), and we use it in this paper to obtain coloured stochastic Petri nets.

Coloured Petri nets can be constructed from uncoloured Petri nets by folding, when partitions of places and transitions are given. These partitions define the colour sets of the coloured net. Vice versa, coloured Petri nets with finite colour sets can be automatically unfolded into uncoloured Petri nets, which then allows the application of all analysis techniques available for the corresponding unfolded net class.

**Fig. 5:**
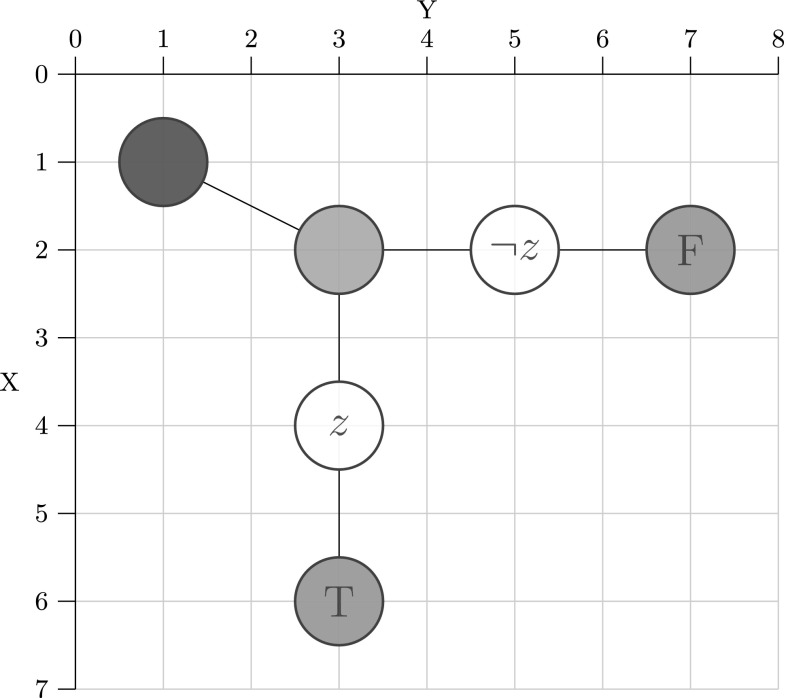
Toy example to illustrate the use of coloured Petri nets. Colour code: blue—INIT, green—FORK, red—FINAL; uncoloured—NORM. (Color figure online)

Coloured Petri nets consist, like standard Petri nets, of places, transitions and arcs. Additionally, a coloured Petri net is characterised by a set of colour sets, and related net inscriptions, which together permit to distinguish tokens by their colours. Defining coloured Petri nets formally would exceed the given space limit; see Liu et al. (2012) for details. Here, we confine ourselves to introduce the essential concepts by means of our application scenario. To illustrate our modelling ideas we use the toy example shown in Fig. 5.

Petri nets can be specified graphically or textually; our tools support both, the latter by use of the Coloured Abstract Net Description Language (CANDL) format (Schwarick et al. 2016). The following description is a combination of both.

#### Encoding the vertices

DNA walkers perform spatially localised computation (Barbot and Kwiatkowska 2015). Colour permits to encode locality, as we have shown in Gilbert et al. (2013). We start with defining a regular rectangular grid; we need seven rows and eight columns for our toy example. The 2D Cartesian coordinates are represented by pairs of colours (integers).

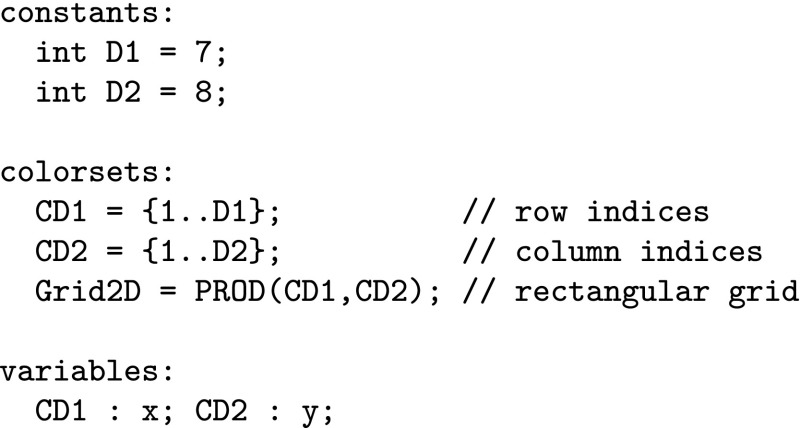



Now, the tuple (*x*, *y*) permits to address the grid element in the x-th row and the y-th column, compare Fig. 5. To encode all attributes of the vertices in the DSD graph, we define two further colour sets of enumeration type.
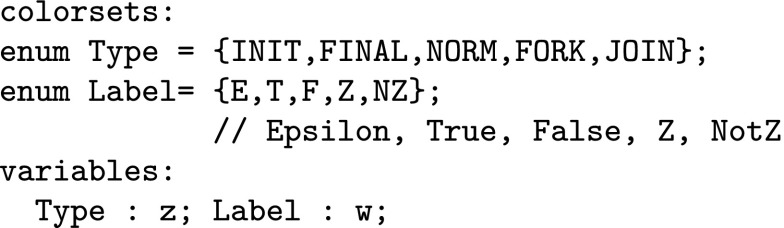


The colour set ${ Label}$ has to be adjusted to ${ literals}(In)$ of a given DSD graph; see Definition 1. Now we have all ingredients to introduce the data type for the vertices, which is a product type over the colour sets ${ CD1}$, ${ CD2}$, ${ Type}$, and ${ Label}.$
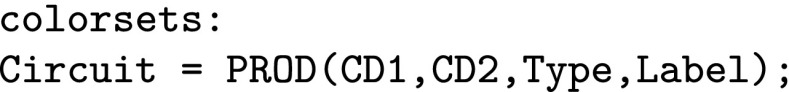


We need two coloured places of this type, *A*—for the anchorages, and *B*—for the blocking mechanism.

The existing vertices with their attributes are defined by the Boolean function *Positions* by enumerating all tuples. Each tuple has to be of the type *Circuit*. Obviously, the definition of this function needs to be adjusted to the given DSD graph. Our toy example has six vertices, so we have six tuples here.
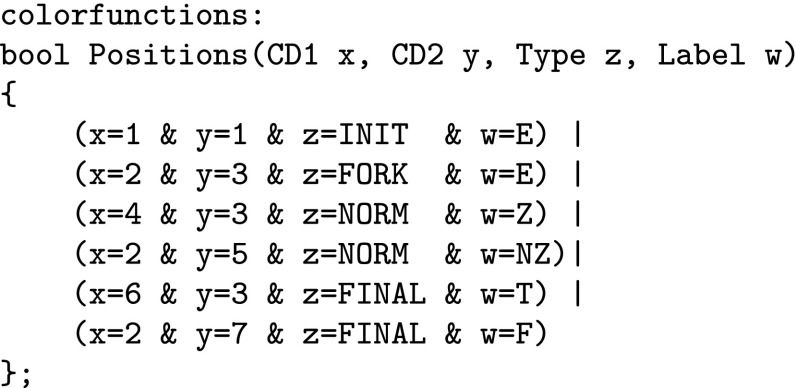


#### Distance metrics

In our modelling approach we are bound to apply a discrete metric, which prevents the use of the popular Euclidean distance. The generalized distance $L_m=\Vert {\cdot }\Vert _m$ between two points $p_1$ and $p_2$ in a plane is defined as

2 $$\begin{aligned} \Vert {p_1 - p_2}\Vert _m = \left( {|}{x_1-x_2}{|}^m + {|}{y_1 - y_2}{|}^m \right) ^{\frac{1}{m}}, \end{aligned}$$

also known as Minkowski distance (Cormen et al. 2001). The rectilinear or Manhattan distance is the $L_1$ distance and is the sum of the absolute differences of the points’ coordinates

3 $$\begin{aligned} \Vert {p_1 - p_2}\Vert _{1} = |{x_1-x_2}| + |{y_1 - y_2}|. \end{aligned}$$

The Euclidean distance is the $L_2$ distance and gives the length of the straight line between two points in Euclidean space

4 $$\begin{aligned} \Vert {p_1 - p_2}\Vert _{2} = \sqrt{\left( x_1-x_2\right) ^{2} + \left( y_1 - y_2\right) ^{2}}. \end{aligned}$$

**Fig. 6:**
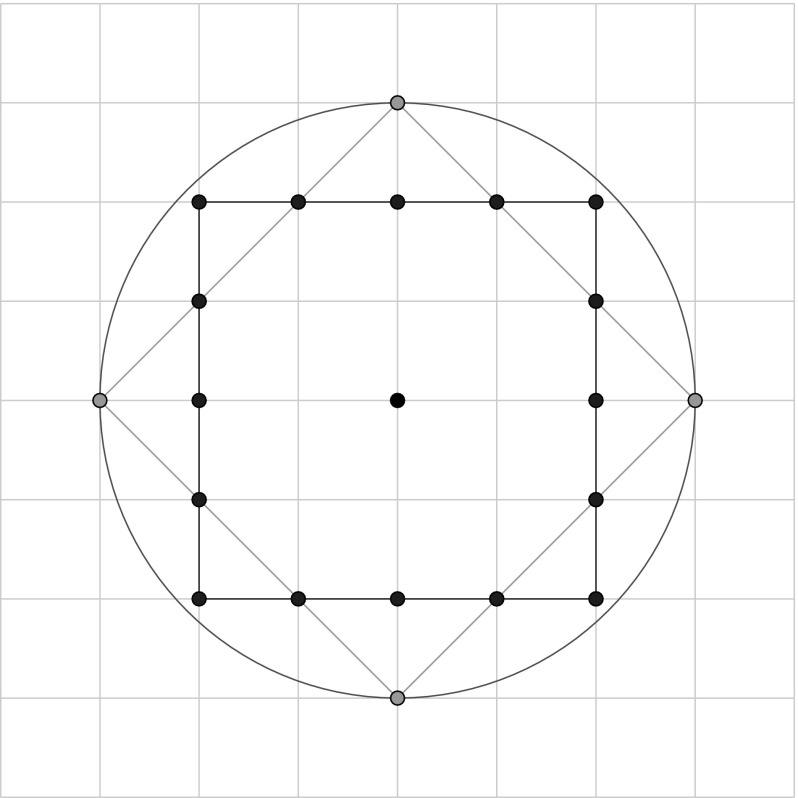
Discrete distance function; $L_1 = 3$ (green) combined with $L_\infty = 2$ (blue) yield together all discrete points within $L_2 = 3$ (red). (Color figure online)

The $L_{\infty }$ distance, also known as Chebyshev or chessboard distance, is the limit of $L_m$ distances for $m\rightarrow \infty$; it is defined as

5 $$\begin{aligned} \Vert {p_1 - p_2}\Vert _{\infty } = { max}\left( |{x_1-x_2}|, |{y_1 - y_2}| \right) , \end{aligned}$$

see Cormen et al. (2001) for details. Equations (3) and (5) yield discrete results for a discrete grid. The combination of the Manhattan distance ($L_1$) and the chessboard distance ($L_\infty$) provides the required results, see Fig. 6, and we define corresponding colour functions; see “Appendix” section for details.

#### Encoding the walker steps

In the following we use the discretised version of Eq. (1):

6 $$k = {\left\{ \begin{array}{ll} k_s & {\text {if}}\quad d \le dS\\ k_s/50 & {\text {if}}\quad dS< d \le dM\\ k_s/100 & {\text {if}}\quad dM < d \le dL\\ 0 & \text {else}, \end{array}\right. } $$

with $dS=3, dM=5, dL=8$. It is obvious how to adjust the resolution of the discretisation to the required precision.

**Fig. 7:**
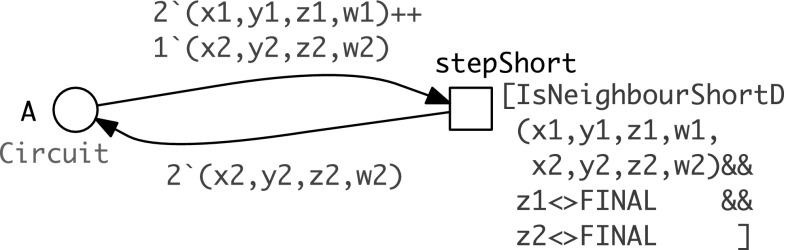
Coloured transition encoding all short distance (regular) steps according to Fig. 3b

We define for each step category

–[short|medium|long] distance [standard|final] steps—a coloured transition and illustrate it here for the short distance standard steps, see Fig. 7. In coloured Petri nets, arcs are weighted with formal sums of tuples: the transition *stepShort* requires two tokens with values bound to (*x*1, *y*1, *z*1, *w*1) and one token with values bound to (*x*2, *y*2, *z*2, *w*2), with the constraint that these two tuples relate to short distance neighbours and none of them is a final vertex. This constraint is expressed as transition guard (given in brackets), which is technically a Boolean expression. A coloured transition can fire for specific values bound to all variables occurring at its adjacent arcs, if its guard is evaluated to true. We introduce the following functions for the transition guard.

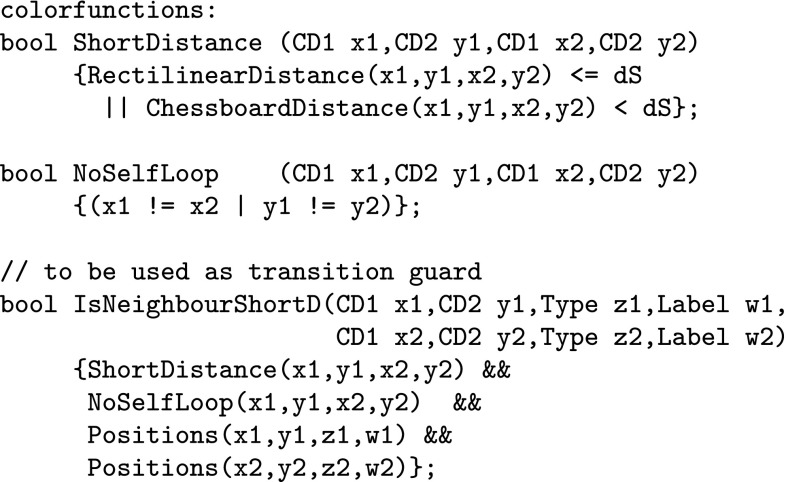



We proceed likewise for the other step categories. Finally, we introduce a coloured transition *loop* which keeps the walker technically alive when having reached a final vertex; see Fig. 12 in “Appendix” section.

#### Blocking of anchorages and fault model

**Fig. 8:**
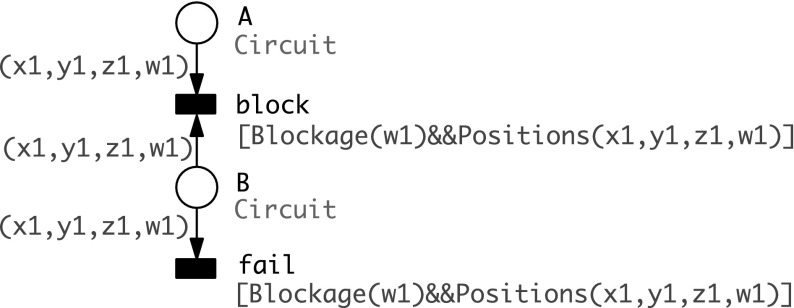
Coloured immediate transitions encoding the blocking and its potential failure for all vertices labelled with ${ Literals}(In)$; compare Fig. 4

Similarly, we introduce coloured transitions encoding the blocking and its potential failure for all vertices labelled with an element of ${ Literals}(In)$. For this purpose, we need a further function:

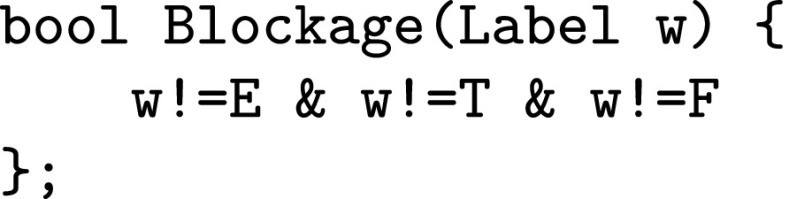



to guard the firing of the two coloured immediate transitions *block* and *fail*; see Fig. 8. Which anchorages have to be blocked for a given set of input values is controlled by the initial marking of the place *B*.

In the appendix, we provide the complete CANDL specification for this toy example. It can be used as template to specify any DNA walker circuit according to Definition 1 as coloured SPN; compare workflow, first step in “Appendix” section. Unfolding these coloured Petri nets generates Petri nets as described in Sect. 2.2. The adaptation of the template to any of the execution semantics discussed there is straightforward.

#### Comparing both Petri net modelling approaches

There is no explicit notion of space in the uncoloured Petri net model, which encodes in a undifferentiated manner the intensional transitions of the DSD graph structure together with medium and long distance relationships. However the functionality of the DNA circuit is also influenced by the 2D topology of the net, for example leakage, and colour permits the construction of multi-dimensional models (Heiner and Gilbert 2013), Thus the coloured Petri net model explicitly contains all locality data, which are exploited in the automated unfolding to generate all transitions according to the defined neighbourhood relations. Using coloured Petri nets, adjusting the model to different distance notions does not require programming skills, and the extension of the approach to the 3-dimensional scenario is straightforward.

More importantly, the use of colour enables the grid layout of the graph and inter-anchorage distance to be directly encoded in the model, and thus leakage can be directly extracted from the model as we will see in the next section.

## Identification of leakage transitions

One of the major issues with existing modelling approaches for DNA walker circuits is the inability to automatically identify leakage transitions. We present an algorithm that investigates the structure of the unfolded Petri net in order to identify leakage transitions.

**Fig. 9:**
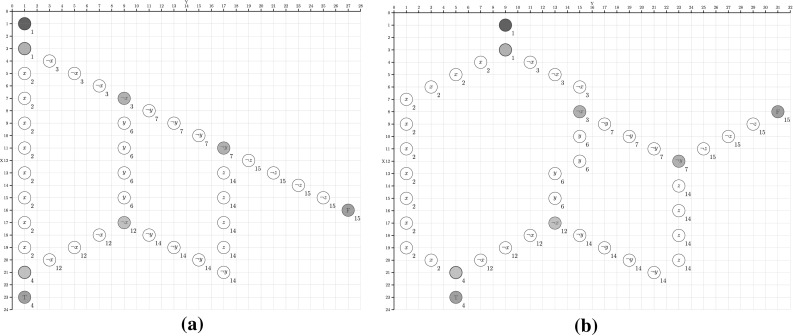
Two layouts for the DSD graph given in Fig. 1 for the Boolean function $x\vee y\vee z$. Both layouts are adapted versions from Dannenberg (2016). The numbers shown next to the vertices are the indices generated by Algorithm 1 for the transition classification. **a** Naive layout, **b** optimized layout

### Place indexing

The underlying idea of the algorithm is to follow the short distance sub-graph (i.e.,the sub-graph comprising only short distance steps), which should unambiguously correspond to the DAG of the intended computation. Any further short distance transitions make the computational DAG ambiguous; these additional transitions may be shortcuts or leakage transitions.

To identify the computational DAG in a given short distance sub-graph we borrow a simple labelling principle widely used to efficiently organise the nodes of a left-complete binary tree in an array data structure (Cormen et al. 2001). Node labels are defined over ${\mathbb {N}}^{+}\,$ and serve as indices in the array, and simple operations over the array indices give direct access to a node’s parent or children nodes; see Fig. 9. We extend this idea to index DAGs, which however makes everything a bit more complicated, as we now have to deal with join vertices as well.

We employ a breadth first search (BFS) over all places of the net, starting with the INIT place that is indexed with 1. Each place that we have to examine is added to a queue, i.e.,a first-in–first-out (FIFO) data structure ensuring a BFS. By indexing the visited places we follow automatically shortest paths to the final places, but with one exception that needs special care, see JOIN places below. To identify the successors of a given place *x*, we introduce a new notation ${x}^{\circ }$, providing the set of post-places *y* of all post-transitions of *x* satisfying

$$\forall y \in {({x}^{\bullet })}^{\bullet } : { index}(y) \not = { index}(x) \wedge [{ index}(y) = 0 \vee { index}(y) \not = { index}(x) / 2].$$

While indexing the places, we collect transition types, which are characterised by triples (pre-node index, post-node index, [TRACK|FORK|JOIN|LEAK]).

Let’s consider the following cases which may occur when indexing an *unambiguous short distance sub-graph*.

- The unique place of type INIT has to have exactly one successor by definition. The successor of INIT gets the same index, i.e.,1, and the successor is added to the queue. The transition from INIT to its successor is part of a (linear) track, thus we add a tuple of (${1,1,{ TRACK}}$) to the known transition types.
- For binary forward branching, a node *c* of type FORK has 3 short distance neighbours, one corresponds to the predecessor and the others to the two branches the computational path may take. The first of the two successors ${c}^{\circ }_0$ gets an index that is two times the index of *c*. Then ${c}^{\circ }_0$ is added to the queue. The second successor ${c}^{\circ }_1$ gets an index that is two times the index of *c* plus one. Then ${c}^{\circ }_1$ is added to the queue. The transitions from *c* to its successors are FORK transitions, so we add two triples (*c*, ${c}^{\circ }_i$, FORK) to the known transition types.
- Binary backward branching takes place on a node *c* of type JOIN and as such *c* has 3 short distance neighbours, two predecessors and one successor. When the algorithm reaches *c*, it may happen that two of the neighbours are not indexed yet. In this case we are not able to decide which one is the predecessor and which one is the successor, so we postpone that decision and treat both places as if they each would be a successor. The successor gets the same index as *c* and is added to the queue. The transitions between *c* and its successor are of type TRACK, i.e.,the tuple of both indices and type TRACK is added to the known transition types.
- A node of type NORM in a linear track has exactly two short distance neighbours, one corresponds to the predecessor, and one to the successor node. But there are several cases to deal with.
  1. The successor is not indexed yet and is of type JOIN. So it gets an index two times of *c*, and the transition must be of type JOIN too. The successor is added to the queue.
  2. The successor is not indexed yet and is not of type JOIN. So it gets the same index as *c*, because it is on the same track. Thus the transition is of type TRACK. The successor is added to the queue.
  3. The index of the successor is smaller than the index of *c* and the successor is of type JOIN. So we have reached an already visited backward branch and add the tuple of indices and type JOIN to the known transition types.
  4. The index of the successor is smaller than the index of *c* and the successor is not of type JOIN. So we override the successor’s index and add it to the queue, because we are backtracking to a previously visited track.
  In each of the previous cases, the transition between *c* and its successor is of type JOIN or TRACK, i.e., a triple of both indices and type JOIN or TRACK is added to the known transition types.
  A normal node may have 3 short distance neighbours in the case of leak transitions. In this situation we have to take care of several cases.
  1. The successor is not indexed yet and is of type JOIN, so it gets an index two times of *c* and the transitions must be of type JOIN too, the successor is added to the queue and is additionally marked.
  2. The successor is not indexed yet and is not of type JOIN, so it gets the same index as *c*, because they are on the same track. Thus the transitions are of type TRACK and the successor is added to the queue and is additionally marked.
  3. The successor is marked and is of type JOIN, so we have reached an already visited backward branch and add a tuple of the indices and type JOIN to the known transition types.
  4. The successor is marked and is not of type JOIN, so we overwrite the successor’s index, if it is smaller, because we reached a corner with shortcut transitions.
  5. The successor is not marked; thus the transition must be a leak transition. The transitions from *c* to its successors are LEAK transitions, so we add two triples (*c*, ${c}^{\circ }_i$, LEAK) to the known transition types.
  In the first four cases, the transitions between *c* and its successor are of type JOIN or TRACK, i.e., tuples of both indices and type JOIN or TRACK are added to the known transition types.
- A node of type FINAL has only one short distance neighbour, which is then a predecessor, so there are no more nodes to investigate.

The algorithm terminates, when the queue is empty and all places are indexed. Furthermore, we obtain a set of tuples defining the known transition types between pairs of indices. In all other cases, the algorithm sends a warning and terminates; see Algorithm 1 for its pseudo code.

### Transition classification

Having indexed the short distance sub-graph, we classify the steps deploying the set of known transition types, i.e., the set *M* computed by Algorithm 1. There are four types of steps: TRACK, FORK, JOIN and LEAK.

- The pre-place and post-place of a TRACK transition *t* have the same index, forming a linear track, i.e.,${ index}({t}^{\bullet }) = { index}({}^{\bullet }t)$.
- For the indices of the pre- and post-place of a FORK transition *t*, it holds either ${ index}({t}^{\bullet }) = 2\cdot { index}({}^{\bullet }t)$ or ${ index}({t}^{\bullet }) = 2\cdot { index}({}^{\bullet }t) + 1$.
- For the indices of the pre- and post-place of a JOIN transition *t*, it holds either ${ index}({t}^{\bullet }) = 2\cdot { index}({}^{\bullet }t)$ or ${ index}({t}^{\bullet }) < { index}({}^{\bullet }t)$.
- There is one precisely defined case of LEAK transitions—a leak that follows directly after a fork. Thus, any transition satisfying ${ index}({t}^{\bullet }) = { index}({}^{\bullet }t) + 1$ or ${ index}({}^{\bullet }t) = { index}({t}^{\bullet }) - 1$ is a LEAK transition. However, depending on the layout, a leak can occur anywhere between two places having different indices.

The classification of steps into TRACK, FORK, JOIN and LEAK takes place by looking up the set *M* of known transition types. If there is a tuple in *M* of the indices of the pre- and post-place (or vice-versa) of the step, then the associated type is allocated to the step. All remaining, non-classified steps have to be leaks and are classified accordingly. In combination with the three step distances (short, medium, long), we are able to provide a concise classification of all transitions into 12 categories. Short distance leakage transitions clearly indicate potential for layout improvement.

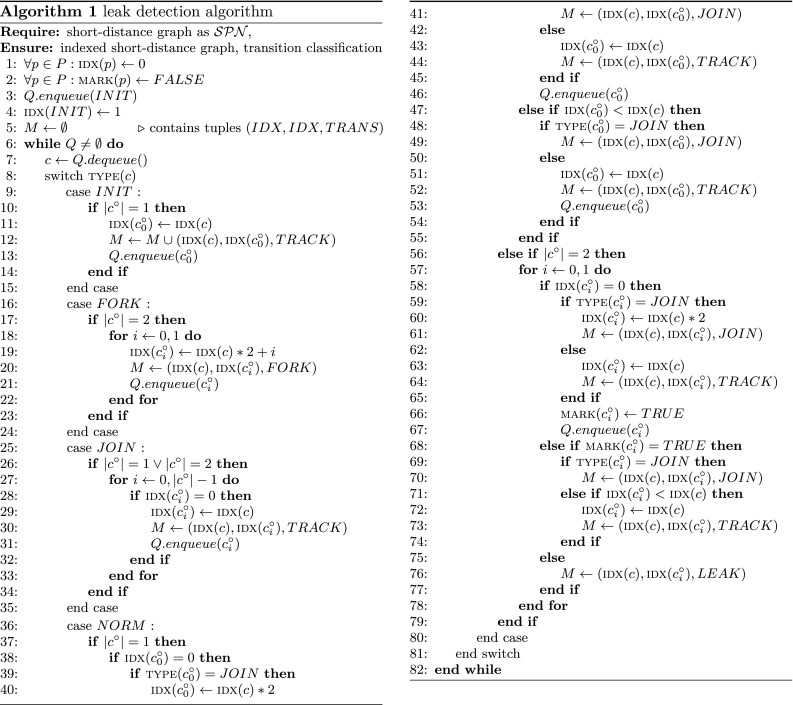



### Case study

For demonstration purposes we use two layouts for the Boolean function $x\vee y\vee z$, which are inspired by Dannenberg (2016). The first layout can be regarded as rather naive and incorporates some flaws resulting in leakage transitions, see Fig. 9a. The second layout is optimised in the sense that the number of leakage transitions is reduced, see Fig. 9b. Tables 1 and 2 show the results for the two layouts of Fig. 9. They confirm that the layout in Fig. 9b is better than the layout in Fig. 9a with respect to leakage transitions. As expected, the number of short distance FORK and short distance JOIN transitions are the same for both layouts.

**Table 1:** Transition classification for the naive layout in Fig. 9a

	Short	Medium	Long	$\varSigma$
Track	64	51	51	166
Fork	12	30	54	96
Join	8	17	33	58
Leak	8	36	98	142

**Table 2:** Transition classification for the optimised layout in Fig. 9b

	Short	Medium	Long	$\varSigma$
Track	66	57	57	180
Fork	12	34	62	108
Join	8	20	30	58
Leak	2	20	58	80

### Implementation

The algorithm presented for indexing an unambiguous short distance sub-graph satisfying Definition 1 enables us to classify step transitions and thereby to identify leakage transitions. It is implemented in our advanced Petri net analysis tool MARCIEand is available under http://www-dssz.informatik.tu-cottbus.de/DSSZ/Software/Marcie.

## Analysis techniques

### Qualitative analysis

We obtain the Petri nets to be analysed by automatic unfolding of coloured SPNs following our template for DNA walker circuit specification. Thus, by construction, we always obtain a very special net class of Petri nets, which correspond—from a behavioural point of view—to finite automata. At any point of time, the walker can be at exactly one anchorage, the walker cannot multiply itself and can never disappear.

To increase our confidence in the template and to deepen our understanding of its behaviour, we apply a popular analysis technique relying on an exhaustive description of all possible behaviour. For this, we compute all markings (system states) reachable from the initial marking $m_{0}$ by any firing sequence of arbitrary length, written as $[ m_0 \rangle$, forming the *state space* of a given Petri net. The reachability relation over the state space is known as the reachability graph.

#### **Definition 4**

(*Reachability graph*) Let ${\mathcal {N}}=({P,T,f,m_0})$ be a Petri net. The reachability graph of ${\mathcal {N}}$ is the graph ${\mathcal {RG(N)}} = \left( V_{{\mathcal {N}}}, E_{{\mathcal {N}}} \right)$, where

- $V_{{\mathcal {N}}} := [m_0 \rangle$ is the set of nodes,
- $E_{{\mathcal {N}}} := \{\, (m, t, m') \mid m, m' \in [m_0 \rangle , t \in T: {m} {\xrightarrow {\text {t}} }{m'} \}$ is the set of arcs. □

The nodes of a reachability graph represent all possible markings of the net. The arcs in between are labelled by single transitions, the firing of which causes the related state change. The reachability graph gives us a finite automaton representation of all possible single step firing sequences. Consequently, concurrent behaviour is described by enumerating all interleaving firing sequences; so the reachability graph reflects the behaviour of the net according to the interleaving semantics.

Generally, reachability graphs tend to be huge. In the worst-case the state space grows faster than any primitive recursive function (Priese and Wimmel 2003). In our case, the size of the state space depends on the total number of vertices and the number of vertices to be blocked. The state space may explode for DNA walker models because all paths have to be generated which a walker can take. Moreover, the blocking and its failure introduces concurrency, which is analysed by considering all interleaving sequences of the transitions generated by unfolding the coloured transitions *block* and *fail*.

If we succeed in constructing the complete reachability graph, we are able to decide behavioural Petri net properties. We recall the most important ones, which include the three orthogonal behavioural properties—boundedness, reversibility, and liveness.

- A Petri net is *k-bounded* iff there is no node in the reachability graph with a token number larger than *k* in any place.
- A Petri net is *reversible* iff the reachability graph is strongly connected.
- A Petri net is *free of dead states* iff the reachability graph does not contain terminal nodes, i.e., nodes without outgoing arcs.
- In order to decide *liveness*, the reachability graph has to be partitioned into strongly connected components (SCC), i.e., maximal sets of strongly connected nodes. A SCC is called terminal if no other SCC is reachable in the partitioned graph. A transition is *live* iff it is included in all terminal SCCs of the partitioned reachability graph. A Petri net is live iff this holds for all transitions.

Our Petri nets are by construction:

- *2-bounded*: an anchorage can be unblocked (1 token), and host the walker (1 token) at the same time; no more moving tokens do exist;
- *not reversible*: which is an immediate consequence of the “burnt-bridges” scenario, causing acyclic reachability graphs;
- generally, *not free of dead states*: a walker can be trapped in a non-final vertex without any neighbouring vertex free to be visited;
- *not live*: while all transition can occur once in some behaviour, none of them will ever have a chance to fire twice in the “burnt-bridges” scenario.

These behavioural properties obviously depend on the applied execution semantics for the given DSD graph, but they are shared by all instances following the same template. For our execution semantics on hand, they coincide with our expectations.

For a given net, we determine these properties by help of Charlie (Heiner et al. 2015) or Marcie (Heiner et al. 2013). Charlie provides a traditional implementation, which works fine up to about 500,000 states (on current computer technique), while Marcie applies symbolic data structures, which substantially postpone the situation where the size of the state space exceeds the available memory.

Having validated our qualitative Petri nets, we are ready for the next step—the stochastic analysis. Standard stochastic Petri nets fulfilling the Markov property share the reachability graph with its underlying qualitative Petri net. Thus, all qualitative properties are still valid, but their interpretation can be refined by taking probabilities into consideration.

Our application scenario requires immediate transitions, which brings us to Generalised Stochastic Petri Nets (GSPN). Immediate transitions always fire with highest priority. With other words, if an immediate transition and a stochastic transition are concurrently enabled, then in the stochastic setting, only one firing sequence is considered (with the immediate transition firing first), while in the qualitative, time-free setting two firing sequences are considered (immediate—stochastic, stochastic—immediate). Consequently, the reachability graph induced by a GSPN is generally a proper subgraph of its underlying qualitative Petri net, which in turn means that a property relying on a given path in the reachability graph may not hold anymore in a specific sub-graph. For example, a dead state, reachable in the qualitative Petri net, is not necessarily reachable in the stochastic setting. In contrast, if the qualitative Petri net is free of dead states, then this holds for the GSPN as well.

To clarify the situation, we need to deploy stochastic analysis techniques, discussed in the next section.

### Stochastic analysis

We analyse the probabilistic behaviour of DNA walker by means of simulative model checking (Rohr 2017). We start with recalling some properties defined in Dannenberg (2016), before extending the analysis by additional properties.

First, we investigate the transient behaviour of the walker circuits, e.g., how likely it is to have reached some state at a certain time point. This can be achieved by probabilistic model checking using the Continuous Stochastic Logic (CSL) (Baier et al. 2000). It is a stochastic adaptation of the Computation Tree Logic (CTL) (Clarke et al. 2001) to formulate properties over Continuous-time Markov Chains (CTMCs).

In the second part of our stochastic analysis we want to observe derived measures, also called reward, cost, observer, gain or bonus. Hence, we add an extra dimension to the CTMC and while moving on in time, it accumulates an output. In order to realise this, a reward structure $(\underline{\rho },{\varvec{\iota }})$ is added to the CTMC. The state reward function $\underline{\rho }:{\mathcal {R}}\rightarrow {\mathbb {R}}^{+}_{0}\,$ defines the rate at which reward $\underline{\rho }(s)$ is obtained in state *s*. That means a reward of $\tau \cdot \underline{\rho }(s)$ is earned, if the CTMC stays in state *s* for $\tau$ time units. The impulse reward function ${\varvec{\iota }}:{\mathcal {R}}\times {\mathcal {R}}\rightarrow {\mathbb {R}}^{+}_{0}\,$ assigns to each transition *t* from state *s* to $s'$ a reward ${\varvec{\iota }}(s,s')$, i.e., a reward ${\varvec{\iota }}(s,s')$ is acquired, if transition *t* fires. Having this, we can perform reward analysis by applying the CSL reward extensions (Kwiatkowska et al. 2007), e.g., what is the expected accumulated reward after some time. For example, rewards can be used to analyse the behaviour of transitions in terms of firing occurrences, which can be accumulated by a class of transitions.

Last but not least, we conduct performability analysis by use of the Continuous Stochastic Reward Logic (CSRL) (Haverkort et al. 2002), which is a superset of CSL. It combines the temporal logic formulas of CSL with a reward function, and the temporal logic operators have an additional reward interval. Now it is possible to reason about the probability to have reached some state at a certain time point and with respect to an interval on the accumulated reward.

#### Transient analysis

We consider four properties for transient analysis and use the same time bound $\tau = 12{,}000\,{\mathrm{s}}$ for all properties, this corresponds to 200 min.

The first property to check is the probability of the walker to have reached any of the FINAL anchorages at time point $\tau$.

7 $$\begin{aligned} {\mathcal {P}}_{=?} \left[ {\text {F}}^{\tau ,\tau } \, {{\mathtt {FINAL}}} \right] \end{aligned}$$

The second property is the probability of the walker to have reached the CORRECT FINAL anchorage according to its input values at time point $\tau$.

8 $$\begin{aligned} {\mathcal {P}}_{=?} \left[ {\text {F}}^{\tau ,\tau }\, {\mathtt {CORRECT}} \right] \end{aligned}$$

The third property is the probability of the walker to get stuck on its way in a dead state. The atomic proposition DEADLOCK describes the set of dead states.

9 $$\begin{aligned} {\mathcal {P}}_{=?} \left[ {\text {F}}^{\tau ,\tau } \, {\mathtt {DEADLOCK}} \right] \end{aligned}$$

The forth property is the conditional probability CONDITION of the walker to have reached the CORRECT FINAL anchorage according to its input values given that it has reached any of the FINAL anchorages at time point $\tau$.

10 $$\begin{aligned} {\mathcal {P}}_{=?} ({\mathtt {CONDITION}} )&= {\mathcal {P}}_{=?} ({\mathtt {CORRECT}} \mid {\mathtt {FINAL}} ) \nonumber \\&= \frac{ {\mathcal {P}}_{=?} \left[ {\text {F}}^{\tau ,\tau } {\mathtt {CORRECT}} \right] }{{\mathcal {P}}_{=?} \left[ {\text {F}}^{\tau ,\tau } {\mathtt {FINAL}} \right] } \end{aligned}$$

#### Reward analysis

Observing derived measures requires the definition of a reward function that extends the CTMC by another dimension. One such measure is the accumulated number of steps *n* taken by a DNA walker on its way from the initial anchorage to a final anchorage. This is a discrete random variable, because the DNA walker randomly chooses steps of different length at each anchorage, except for the final anchorage. The number of steps a walker takes on its path can be computed by the impulse reward function ${\varvec{\iota }}_{{ steps}}$ defined by the following reward structure:

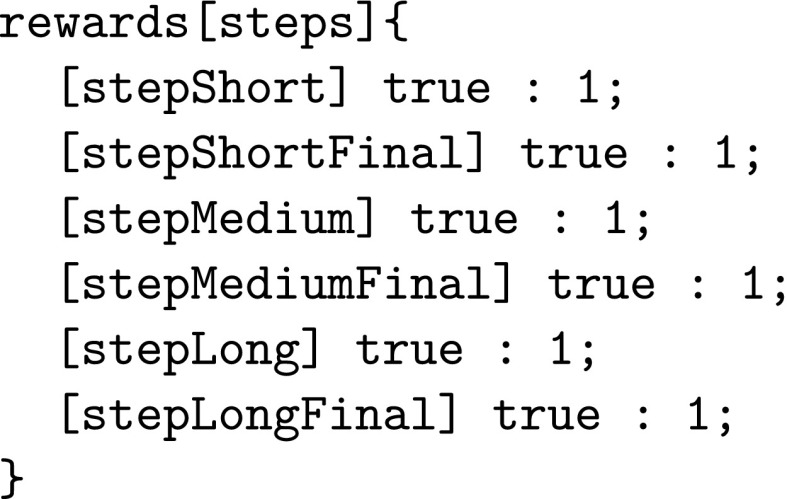



The impulse reward function ${\varvec{\iota }}_{{ steps}}$ is increased by one each time a stepping transition is fired and thus computes the number of steps a walker takes. The expected (average) number of steps of the DNA walker within $\tau = {12{,}000}\,{\mathrm{s}}$ can be computed with the CSL formula

11 $$\begin{aligned} {\mathcal {R}}\{{ steps}\}_{=?} \left[ {\text {C}}^{\le \tau } \right] . \end{aligned}$$

The classification of transitions reveals 12 different classes in the walker circuit under study, e.g., *track-short*, *fork-medium*, *leak-long*, etc.; and we are able to observe them directly. The number of steps of a certain class a DNA walker takes on its path can be computed by the impulse reward function ${\varvec{\iota }}_{{\ll }{\mathrm{class}}{\gg }}$ defined by the following reward structure:

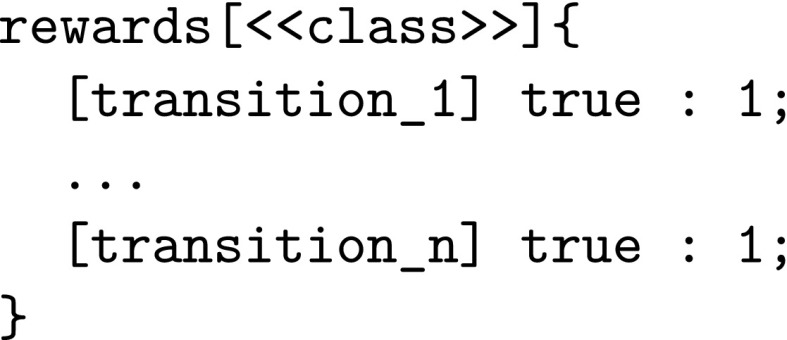



We define one transition reward structure entry per classified transition identified with Algorithm 1. Thus the expected (average) number of classified steps the walker takes within $\tau = {12{,}000}\,{\mathrm{s}}$ can be computed with the CSL formula

12 $$\begin{aligned} {\mathcal {R}}\{{\ll }{\mathrm{class}}{\gg }\}_{=?} \left[ {\text {C}}^{\le \tau } \right] . \end{aligned}$$

#### Performability analysis

The reward analysis reveals the expected number of steps or leakage steps, but we are interested in the probabilities for different numbers of steps or leakage steps, too. Such probabilities are known as performability. We are able to compute the probability distribution of the related random variable deploying simulative model checking of CSRL in combination with impulse rewards (Rohr 2017). We compute the probability to reach a FINAL anchorage within $\tau = {12{,}000}\,{\mathrm{s}}$ by taking exactly *n* steps with the following CSRL formula

13 $$\begin{aligned} {\mathcal {P}}\{{ steps}\}_{=?} \left[ {\text {F}}^{\tau ,\tau }_{n,n} {\mathtt {FINAL}} \right] . \end{aligned}$$

Equation (13) defines the probability mass function (PMF) for the discrete probability distribution of the discrete random variable *n*.

The probability to reach a FINAL anchorage within $\tau = {12{,}000}\,{\mathrm{s}}$ and by taking at most *n* steps can be computed by the CSRL formula

14 $$\begin{aligned} {\mathcal {P}}\{{ steps}\}_{=?} \left[ {\text {F}}^{\tau ,\tau }_{0,n} {\mathtt {FINAL}} \right] . \end{aligned}$$

Equation (14) is the cumulative distribution function (CDF) for the discrete probability distribution of the discrete random variable *n*.

Exchanging the used reward function from ${\varvec{\iota }}_{{ steps}}$ to ${\varvec{\iota }}_{{\ll }{\mathrm{class}}{\gg }}$ allows us to compute the PDF

15 $$\begin{aligned} {\mathcal {P}}\{{\ll }{\mathrm{class}}{\gg }\}_{=?} \left[ {\text {F}}^{\tau ,\tau }_{n,n} {\mathtt {FINAL}} \right] \end{aligned}$$

and the CDF

16 $$\begin{aligned} {\mathcal {P}}\{{\ll }{\mathrm{class}}{\gg }\}_{=?} \left[ {\text {F}}^{\tau ,\tau }_{0,n} {\mathtt {FINAL}} \right] \end{aligned}$$

of the discrete random variable *n* for each class of steps, i.e., the number of steps of each class a DNA walker takes up to time point $\tau = {12{,}000}\,{\mathrm{s}}$.

#### Case study

At first we compute the transient probabilities of Eqs. (7)–(10) for the two layouts. The results of the transient analysis are shown in Table 3. It turned out that both layouts perform equally well in the transient analysis. This leads to the supposition that achieving the reduced number of leak transitions is bought at the cost of a higher probability of reaching a dead state, due to the longer tracks.

**Table 3:** Transient probabilities averaged over all possible input values for the naive layout in Fig. 9a and the optimised layout in Fig. 9b

	Final (%)	Correct (%)	Deadlock (%)	Condition (%)
Naive	68.66	62.93	9.64	91.66
Optimised	59.48	54.53	13.11	91.68

Second we compute the expected number of steps according to Eq. (11) for the two layouts in Fig. 9. For the naive layout, the expected number of steps of one path averaged over all possible input values is 15.59, while it is for the optimised layout 16.02. The slightly higher value for the optimised layout can be explained by the longer tracks and the higher number of vertices.

Next we report the results for expectation analysis exploiting rewards for each class of steps according to Eq. (12). Tables 4 and 5 show the results, which are in-line with the static analysis in Tables 1 and 2. In the naive layout, a short distance leakage transition occurs in the average in every second run, while for the optimised layout, it occurs about in 1 run out of 10. When we look on leakage transitions in total, then we have in the naive layout in average about 1 leakage transition per run, which drops for the optimised layout below 1 leakage transition in every second run.

**Table 4:** Expected reward averaged over all possible input values for the naive layout in Fig. 9a

	Short	Medium	Long	$\varSigma$
Track	9.897	0.282	0.202	10.354
Fork	1.694	0.163	0.172	2.029
Join	1.208	0.139	0.168	1.515
Leak	0.483	0.189	0.311	0.983

**Table 5:** Expected reward averaged over all possible input values for the optimised layout in Fig. 9b

	Short	Medium	Long	$\varSigma$
Track	10.693	0.354	0.253	11.300
Fork	1.669	0.183	0.206	2.058
Join	1.286	0.170	0.170	1.626
Leak	0.114	0.135	0.195	0.444

**Fig. 10:**
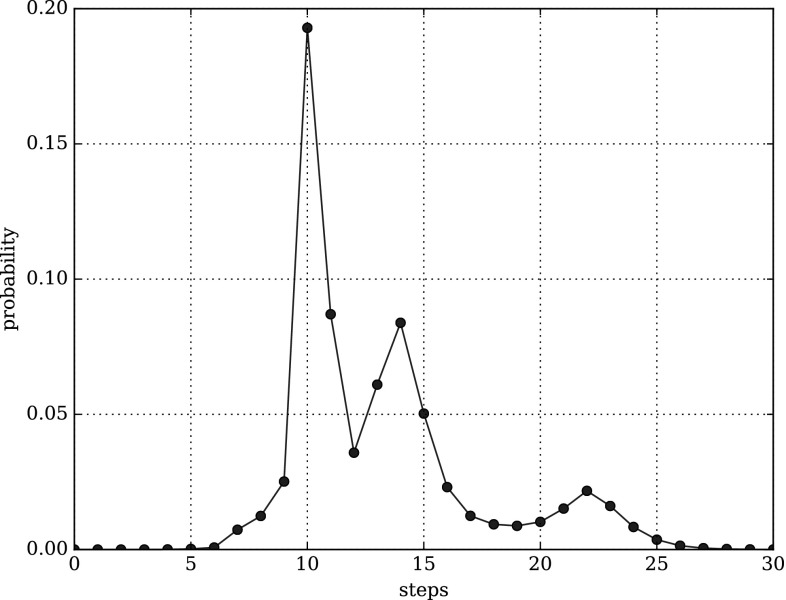
Probability distribution of Eq. (13) computed for $n = (0, 30)$ and the naive layout

**Fig. 11:**
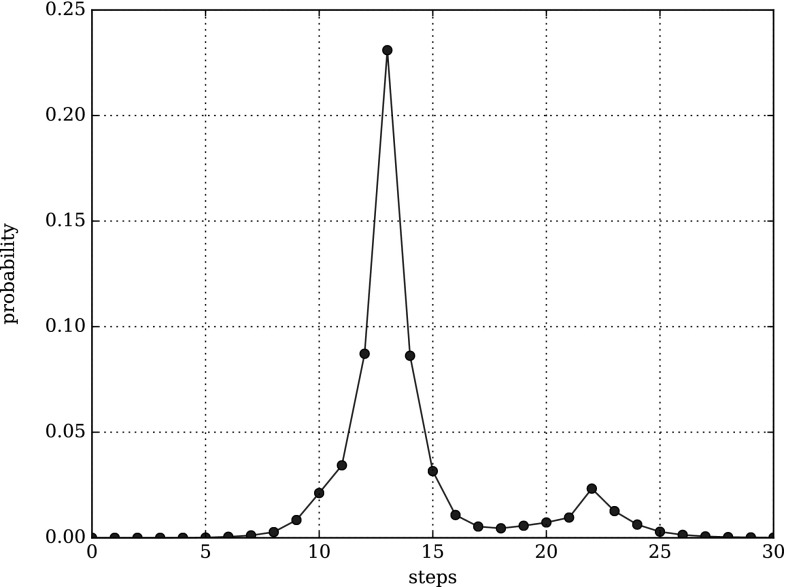
Probability distribution of Eq. (13) computed for $n = (0, 30)$ and the optimised layout

**Fig. 12:**
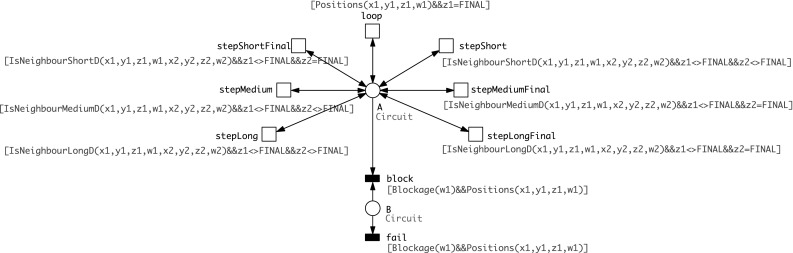
Graphical representation of the coloured SPN; many details not shown, e.g., arc inscriptions, initial marking, transition rates; see CANDL code for all details

The last point in our stochastic analysis is the computation of the performability for the overall number of steps needed to reach a FINAL anchorage. Therefore, we check Eq. (13) for several values of *n*. Figures 10 and 11 show the probability distributions for both layouts. The second peak in Fig. 10 and its wider curve suggest a higher variability in the number of steps required to reach a FINAL anchorage in the naive layout, while the narrow, almost bell-shaped curve in Fig. 11 suggests a more constant number of steps in the optimised layout. This difference in the variability may be caused by the different numbers of leakage transitions.

#### Design trade-off

The results of structural and probabilistic analysis illustrate that we are facing an optimization problem (as discussed in terms of ‘Design principles’ in Dannenberg (2016)):

- *Objective 1*: reduce number of leakage transitions (as more leakage transitions increase the probability of getting the wrong result).
- *Objective 2*: reduce length of tracks (as longer tracks increase the deadlock probability).

Both objectives contradict each other: number of leakage transitions are reduced by increasing the length of tracks; and vice versa; so the challenge in circuit design is to find the right balance between both objectives. Compared with Dannenberg (2016), we are able to quantify objective 1 by a structural analysis. In future work, it would be interesting to explore if also objective 2 could be quantified by a structural analysis (e.g.,total length of shortest linear paths), to obtain a cheaper circuit design assessment.

## Conclusions

In this paper we have reported a novel technique for the 2D modelling of DNA walker circuits using coloured stochastic Petri nets which enables functionality, topology and dimensionality all to be integrated in one two-dimensional model.

The move to coloured Petri nets not only brings a concise representation, but even more importantly a high degree in flexibility with respect to the topology of the anchorages and distance measures of the walker steps, both can be adjusted on the modelling level, no programming required. The anchorages to be blocked are automatically derived from the given input values (true or false) for all parameters of the given Boolean function, this is less error prone than setting them manually.

In terms of technology, the coloured approach enables construction of concise templated models which can be robustly expanded using standard mechanisms built into coloured Petri net tools. Other approaches require individually handcoded programs for translating circuit descriptions into SPN models (Barbot and Kwiatkowska 2015) or PRISM models (Dannenberg et al. 2015).

The concept of immediate transitions as in Petri nets does not exist in the PRISM language, thus it has to be approximated by very high transition rates, which increase the stiffness of the system and may cause numerical issues.

Automatic identification of leakage transitions is a relevant problem, which has remained unsolved until this research. We classify transitions between anchorages into short, medium and long distance categories, which enables a fine-grained analysis of the behaviour of the model. We present an algorithm for the automatic identification of leakage transitions, exploiting the unfolding of the coloured Petri net model. Leakage transitions are classified according to the used distance measure. Our algorithm is innovative, flexible and works for any kind of topologies and distance measures. The identification/classification of leakage transitions is merely a qualitative analysis technique and thus less expensive than CTMC based analysis. We show how advanced stochastic analysis including impulse rewards and performability analysis based on simulative CSRL model checking can be deployed to explore the stochastic behaviour of DNA circuit models. To the best of our knowledge this technique is not supported by other tools so far.

We illustrate the application of these techniques to compare the performance of two alternative layouts for an example DNA walker circuit. The results confirm that leakage can be reduced by employing a circuit layout topology that increases the distance between any two anchorages potentially permitting leakage transitions. An implication of this is that leakage reduction involves increasing the area of the circuit for a given number of anchorages. Since one goal of DNA circuit design is to minimise circuit area, the ability to identify leakage transitions is an important step in the process of optimising DNA circuit layouts, taking into account minimisation of both the computational error and area of circuits. Moreover, the use of multi-dimensional models opens the way to multi-dimensional model checking along the lines of Pârvu and Gilbert (2014).

## Figures and Tables

**Fig. 13:**
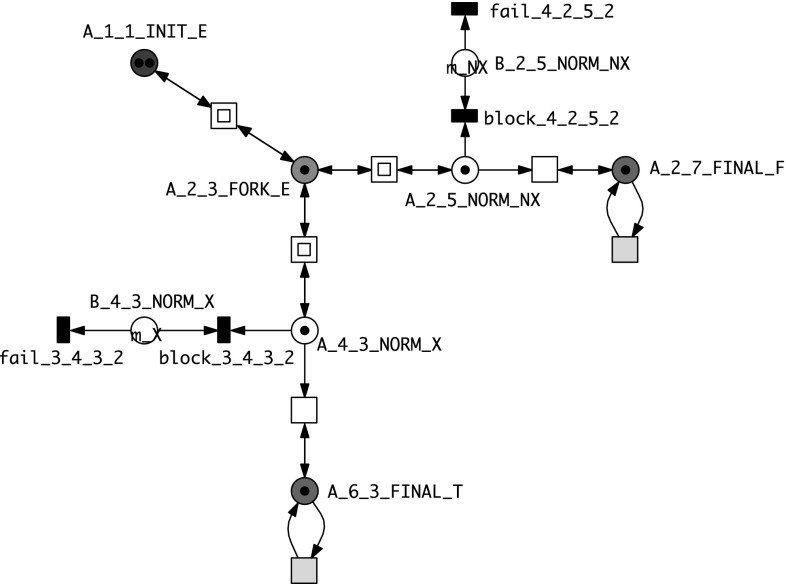
Petri net generated by unfolding a CANDL specification containing only short distance steps; transition names and arc weights not shown. Colour code: places—as used in Fig. 5, yellow transitions—loop, added for technical reasons, see Sect. 4.1. (Color figure online)

**Fig. 14:**
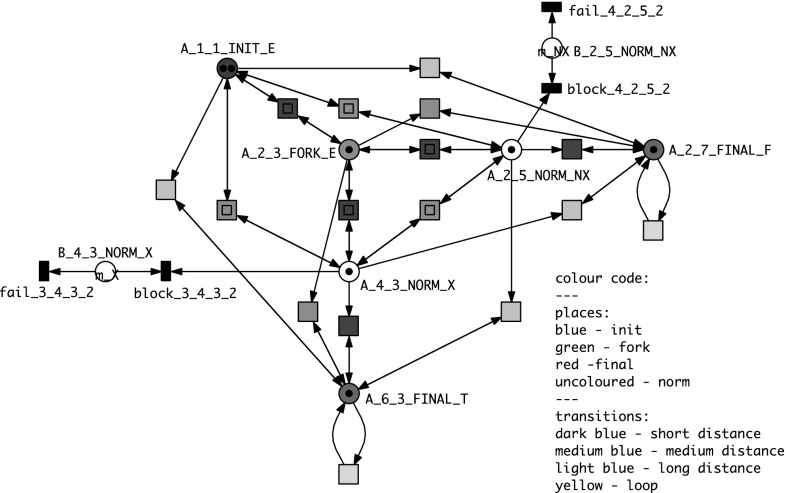
Petri net generated by unfolding the CANDL specification; transition names and arc weights not shown

## Appendix

This appendix provides a full documentation of the toy example, see Fig. 5, employed in Sect. 2.3 to explain the use of coloured Petri nets for the specification of DNA walker circuits. All files can be found at http://www-dssz.informatik.tu-cottbus.de/DSSZ/Software/Examples, and the software tools required at http://www-dssz.informatik.tu-cottbus.de/DSSZ/Software/Software.

### Workflow

Our workflow deploys the following tools:

- MARCIE (Heiner et al. 2013)—leakage detection, qualitative and stochastic analysis;
- Snoopy (Heiner et al. 2012)—graphical design, visualisation and simulation;
- Charlie (Heiner et al. 2015)—structural and qualitative analysis.

If you want to reproduce one of our examples or try your own ones, please follow these steps.

1. Write with your preferred text editor a CANDL specification. Start from the template provided, and adjust
  - the definition of constants in the group *block* according to the ${ literals}(In)$ of your DSD graph, see Definition 1, and the input values;
  - the colour set ${ Label}$ to include ${ literals}(In)$;
  - the colour function *Positions* to define all vertices of your DSD graph.
  That’s it!
2. This CANDL specification can be processed by Marcie
  - to determine the leak transitions, categorised into short/medium/long distance transitions:
  - to compute the state space, if possible, to determine dead states;
  - for any stochastic analysis as described in Sect. 4.2.
3. Alternatively, unfold the CANDL specification with  to obtain an ANDL file. This step is only mandatory if one wants to employ Charlie.
4. This ANDL file can be processed by our tools. Use
  - Sooopy—to obtain a graphical representation of the unfolded net: $\rightarrow$ file $\rightarrow$ import
  - Marcie—to determine the leak transitions, categorised into short/medium/long distance transitions:
    *Remark* The Petri net provided as andl file has to be obtained by unfolding a coloured SPN following our template.
  - Charlie—for structural analysis;
  - Marcie—to compute the state space, if possible, to determine the dead states;
  - Marcie—for any stochastic analysis as described in Sect. 4.2.

Please see Marcie’s Manual for more details (Schwarick et al. 2016).

### Coloured SPN: CANDL specification

For reasons of completeness we provide a graphical representation of the coloured SPN template, made by reading the CANDL code into Snoopy; see Fig. 12. Many details are hidden in the graphics; the following CANDL code provides all details. See the Marcie manual (Schwarick et al. 2016) for a formal definition of the CANDL syntax.

### Generated SPN: graphics

The graphical representations shown in Figs. 13 and 14 take advantage of a special feature supported by Snoopy allowing for hierarchical Petri net design where *macro transitions* (represented by two centric squares) stand for subnets. Here we use these transitions to hide the details of the Petri net representation for an undirected step according to the “burnt-bridges” scenario, see Fig. 3b.
